# FOXM1 facilitates gastric cancer cell migration and invasion by inducing Cathepsin D

**DOI:** 10.18632/oncotarget.19254

**Published:** 2017-07-15

**Authors:** Li Yang, Ming Cui, Liang Zhang, Lei Song

**Affiliations:** ^1^ Department of Intensive Care Unit, The Second Affiliated Hospital of Dalian Medical University, Dalian 116027, Liaoning, China; ^2^ Department of Emergency, The Second Affiliated Hospital of Dalian Medical University, Dalian 116027, Liaoning, China; ^3^ Department of Interventional Therapy, The Second Affiliated Hospital of Dalian Medical University, Dalian 116027, Liaoning, China

**Keywords:** FOXM1, Cath-D, gastric cancer

## Abstract

Forkhead box M1 (FOXM1) has been reported as a vital transcription factor in different human malignancies. To date, the mechanisms of FOXM1 in modulating the invasion and metastasis of gastric cancer cells have not been elucidated. In the present study, we found that overexpression of FOXM1 prompted cell migration and invasion of gastric cancer, and increased the expression of Cathepsin D (Cath-D). However, FOXM1 siRNA repressed cell migration and invasion, and also decreased the expression of Cath-D in gastric cancer cells. Blocking of Cath-D repressed FOXM1 overexpression-mediated cell migration and invasion. Mechanically, FOXM1 facilitated the activation of Cath-D promoter. Furthermore, overexpression of Cath-D affected the expression of E-cadherin, leading to epithelial-mesenchymal transition (EMT) of gastric cancer cells. In conclusion, this study demonstrated that FOXM1 promotes gastric cancer cell migration and invasion through inducing expression of Cath-D in gastric cancer. Thus, FOXM1 may be recommended as a potential therapeutic target for gastric cancer patients.

## INTRODUCTION

Gastric cancer is the most common one in cancer-caused deaths in this world. Surgical operation and adjuvant chemotherapy have been demonstrated to be effective in the early stages of gastric cancer [1, 2]. Unfortunately, patients with gastric cancer often developed post-operative recurrence and lymph node metastasis. Overall, the 5-year overall survival rate remains very low, and the effects of present treatment options are not satisfying [3–5]. Thus, it is essential to figure out the complex molecular mechanisms underlying invasion and metastasis of gastric cancer to identify novel, useful markers and targets for the treatment of gastric cancer.

It has been reported that the phosphoinositide 3-kinase (PI3K)/protein kinase B (PKB, also called AKT) pathway was involved in the development of gastric cancer [6]. Notably, the forkhead/winged helix box class O (FOXO) transcription factors act as downstream targets of the PI3K/PKB pathway. In humans, the FOXO family consists of four members, includingFOXO1, FOXM1, FOXO4 and FOXO6, which induce or suppress some genes related to biological functions, involving cell proliferation, apoptosis, metastasis, resistance to stress and DNA repair [7–9]. Increasing reports showed that FOXM1 might facilitate the growth and metastasis of cancer cells. As reported, FOXM1 promotes cancer cell survival by activating NF-κB expression under stress conditions [10–13]. In addition, FOXM1was reported to accelerate metastasis of colon cancer [14]. Thus, FOXM1 is likely to be used as an effective target for cancer patients [15]. However, the role and mechanism of FOXM1 has not been elucidated completely in gastric cancer.

In the previous study, we assumed that FOXM1 might be a potential cancer inducer in gastric cancer. We tried to explore potential target genes of FOXM1 involved in cell invasion and migration. And then we used luciferase assays and western blot analysis to identify Cath-D as a target gene of FOXM1. Eventually, we knocked down endogenous Cath-D using its siRNA to elucidate the role of FOXM1 in inducing tumor development. Our study will lay a new foundation for a potential therapeutic target for gastric cancer patients.

## RESULTS

### Expression of FOXM1 in gastric cancer cell lines

**Figure 1:**
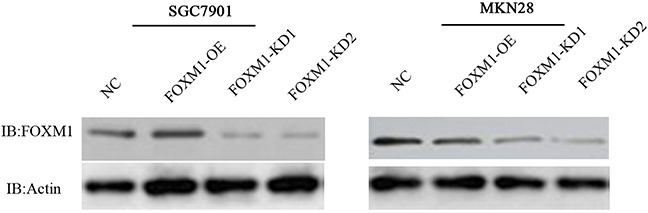
Knockdown and overexpression of FOXM1 in gastric cancer SGC7901 and MKN28 cell lines Western blot analysis was used to detect the expression of FOXM1 in SGC7901 and MKN 28 cells with FOXM1 knockdown (KD), overexpression (OE) or negative control (NC). Western blot analysis demonstrated that FOXM1 expression was obviously decreased in the knockdown (KD) cell lines but markedly up-regulated in the overexpression (OE) cells as compared with their respective controls.

To explore the role and mechanisms of FOXM1 in the progression of gastric cancer, we knocked down and overexpressed FOXM1 using their vectors in both gastric cancer cell lines, SGC7901 and MKN28 (Figure 1). Western blot analysis demonstrated that FOXM1 expression was obviously decreased in the knockdown (KD) cell lines but markedly up-regulated in the overexpression (OE) cells as compared with their respective controls.

### FOXM1 promotes cell migration of gastric cancer

**Figure 2:**
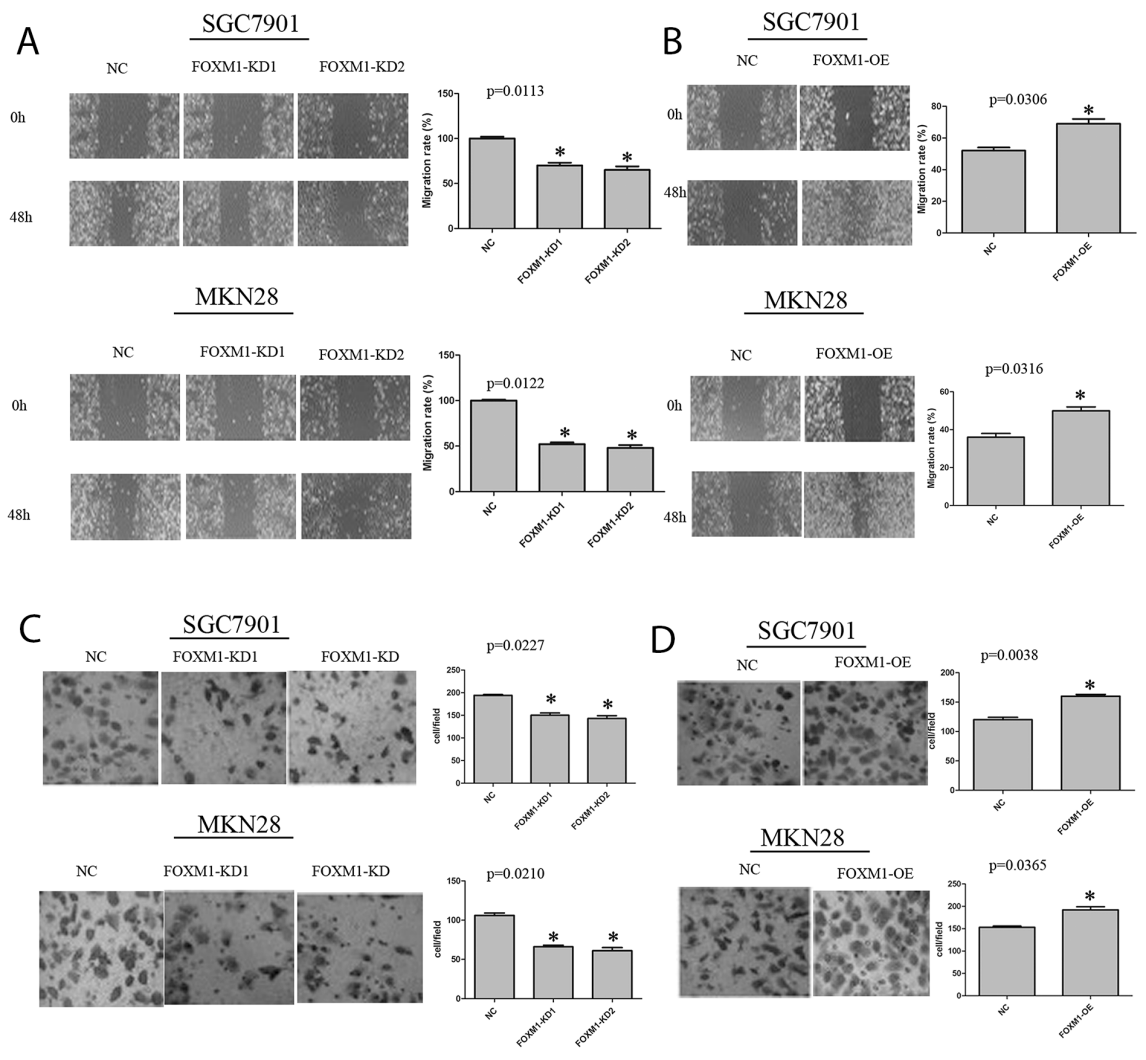
Migration and invasion of SGC7901 and MKN28 cells with FOXM1 knockdown or overexpression **(A)** Depletion of FOXM1 in SGC7901 and MKN28 cells resulted in a significant decrease in wound healing. **(B)** Over-expression of FOXM1 in SGC7901 and MKN28 cells resulted in a significant increase in would healing. **(C)** Depletion of FOXM1 in SGC7901 and MKN28 cells resulted in a significant decrease in invasive migration. **(D)** Over-expression of FOXM1 in SGC7901 and MKN28 cells resulted in significant increase in invasive migration. Images are representative of three independent experiments. Values are Mean ± SEM. *denotes significance at p<0.01 relative to control by student t-test.

To analyze the impact of FOXM1 on cell migration of both gastric cancer SGC7901, and MKN28 cell lines, we performed wound-healing assays. As illustrated in Figure 2, the wound healing assay revealed that knockdown of FOXM1 in both cells led to an obvious reduction in wound healing at 48 hours, while overexpression of FOXM1 in both cells led to an obvious increase in the wound healing.

### FOXM1 promotes cell invasion of gastric cancer cells

**Figure 3:**
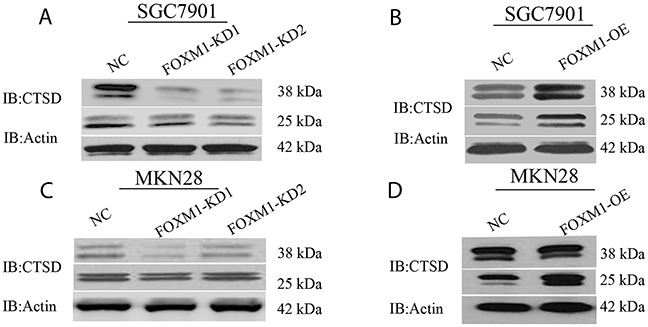
Cath-D expression in FOXM1 knockdown or overexpressing SGC7901 and MKN28 cells Western blot assay was used to detect Cath-D (CTSD) expression in SGC7901 **(A, B)** and MKN28 cells **(C, D)** with FOXM1 knockdown and overexpression, probed with antibody to β-actin.

**Figure 4:**
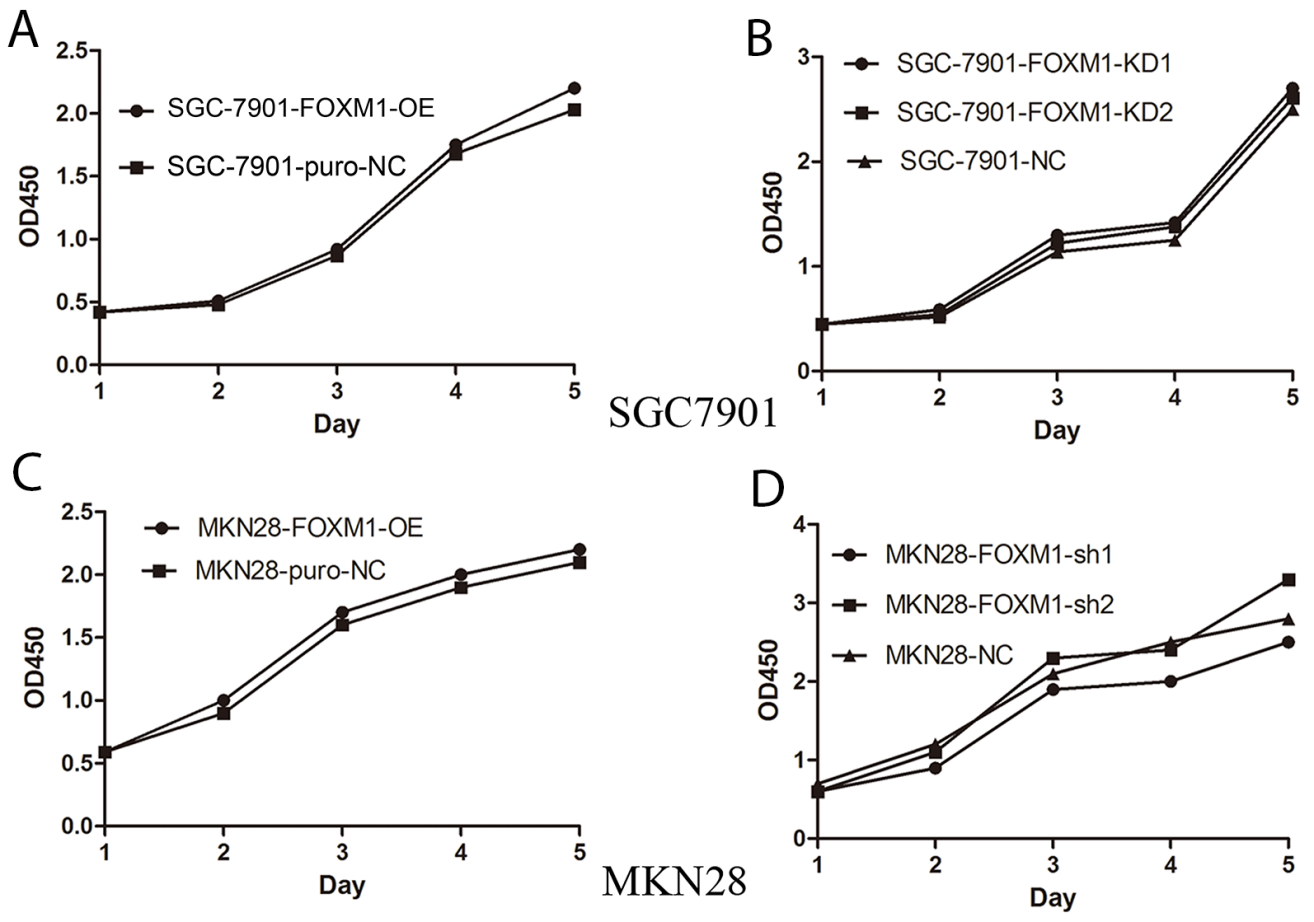
No effects of FOXM1 on proliferation of gastric cancer SGC7901 and MKN28 cells Cell growth of SGC7901 **(A, B)** and MKN28 **(C, D)** was measured using the MTT assay at various time points. Silencing or overexpressing FOXM1 has no effect on cell proliferation compared to negative controls. *denotes significance at p<0.01 relative to control by student t-test.

To further figure out the function of FOXM1 in tumor invasion and metastasis, we detected migration and invasion of both cell lines using standard Matrigel chemoinvasion assays. As shown in Figure 3, knockdown of FOXM1 obviously decreased cell invasion, while overexpression of FOXM1 significantly increased cell invasion. Notably, FOXM1 showed no effects on cell proliferation of SGC7901 and MKN28 cells (Figure 4).

### FOXM1 mediates of Cath-D expression in gastric cancer cells

Since Cath-D plays a crucial role in degradation of extracellular matrix (ECM) and tumor invasion, we further identified whether FOXM1 modulates of Cath-D expression using western blot analysis in SGC7901 and MKN28 cells. As shown in Figure 3, western blot analysis revealed that cells with knockdown of FOXM1 exhibited a decrease in mature Cath-D expression. On the other hand, cells with overexpression of FOXM1 showed an increase in the expression of mature Cath-D, suggesting that Cath-D might be a direct transcriptional target of FOXM1.

### The expression of Cath-D and FOXM1 in gastric cancer tissues

**Figure 5:**
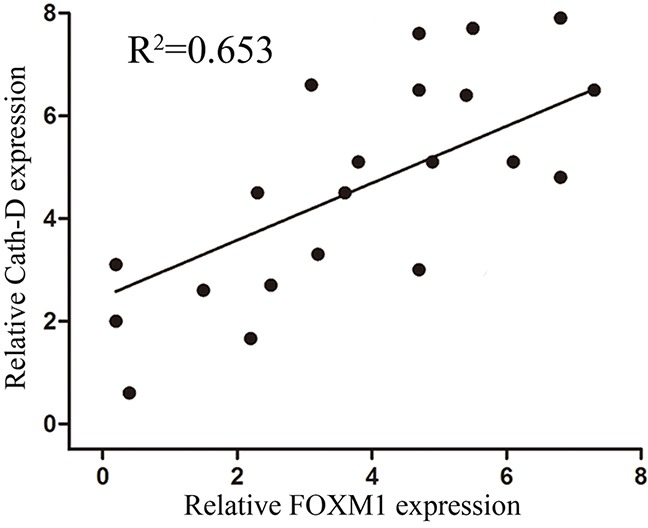
Expression of FOXM1 and Cath-D in gastric cancer tissues and their correlations Bivariate correlation analysis of the relationship between FOXM1 and Cath-D expression level (R^2^=0.653, p=0.011).

Based on results above, we further analyzed the expression of FOXM1 and Cath-D proteins in 20 cases of gastric cancer tissues using immunohistochemistry (IHC). IHC analysis revealed that 70.2% of gastric cancer tissues with high Cath-D expression. Tissues with high Cath-D expression also exhibit high FOXM1 expression (Figure 5). Spearman correlation analysis revealed that Cath-D expression was obviously positively associated with FOXM1 expression in gastric cancer tissues (R2=0.653, p=0.011). These findings suggested that FOXM1 might mediate the generation of Cath-D to induce cell invasion and metastasis in gastric cancer.

### FOXM1 induces Cath-D expression to promote migration and invasion in gastric cancer cells

**Figure 6:**
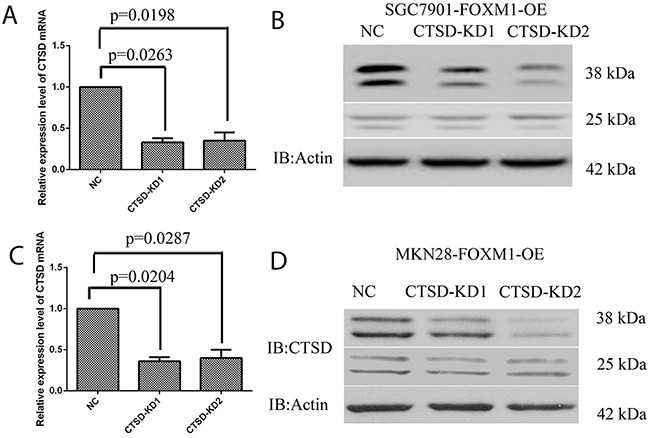
Knockdown of Cath-D in gastric cancer SGC7901 and MKN28 cells **(A, B)** Real-time PCR and western blot analysis of Cath-D expression levels in SGC7901 cells with FOXM1 overexpressing and Cath-D knockdown (KD) or negative control (NC). **(C, D)** Real-time PCR and western blot analysis of Cath-D expression levels in MKN28 cells with FOXM1 overexpressing and Cath-D knockdown (KD) or negative control (NC). *denotes significance at p<0.01 relative to control by student t-test.

**Figure 7:**
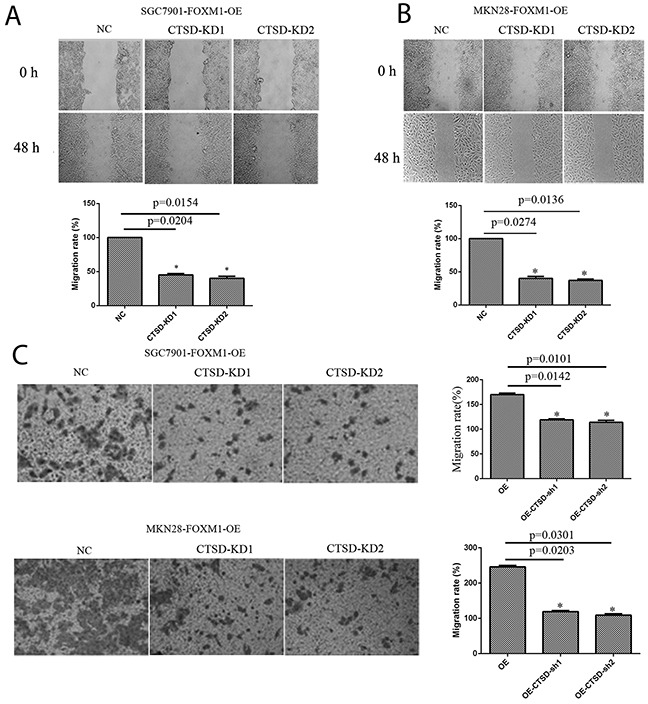
Migration and invasion of SGC7901 and MKN28 cells with FOXM1-OE and Cath-D-KD The FOXM1 overexpressing cells with Cath-D knockdown exhibited decreased cell migration **(A, B)** and invasion **(C)** compared with those FOXM1 overexpressed alone. Images are representative of three independent experiments. Values are Mean ± SEM. *denotes significance at p<0.01 relative to control by student t-test.

**Figure 8:**
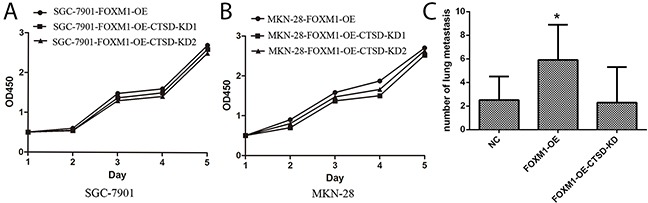
**(A, B)** No effects of Cath-D on proliferation of gastric cancer SGC7901 and MKN28 cells. Cell growth of SGC7901 (A) and MKN28 (B) in which FOXM1 was overexpressed was measured using the MTT assay at various time points. Silencing Cath-D has no effect on cell proliferation compared to negative controls. **(C)** FOXM1 promotes gastric cancer SGC7901 cell metastasis *in vivo*. The numbers of visible metastatic foci in lung of individual mouse. Values are Mean ± SEM. *denotes significance at p<0.01 relative to control by one way ANOVA. Values are Mean ± SEM. *denotes significance at p<0.01 relative to control by one way ANOVA.

To investigate whether Cath-D affected FOXM1-induced migration and invasion in gastric cancer cells, Cath-D siRNA was transfected into SGC7901 and MKN28 cells with FOXM1-overexpression (Figure 6). As illustrated in Figure 7, Cath-D knockdown significantly decreased cell migration and invasion as compared with si-control in SGC7901 and MKN28 cells with FOXM1-overexpression. These findings showed that Cath-D depletion was able to abrogateFOXM1-induced cell migration and invasion. However, knockdown of Cath-D showed no obvious effect on cell growth, indicating that Cath-D was not involved in the processes of cell proliferation (Figure 8A, 8B).

### FOXM1 induces cancer cell metastasis *in vivo*

To investigate the impact of FOXM1 and Cath-D on cancer cell metastasis, we injected FOXM1-OE SGC7901, FOXM1-OE and Cath-D KD SGC7901 and their respective control into subcutaneous tissues of nude mice. In-vivo assay showed that visible metastatic foci or nodules can be obviously observed in the lung of mouse with FOXM1 overexpression alone. However, few metastatic foci or nodules can be found in the lung of mouse with FOXM1 overexpression and Cath-D knockdown (Figure 8C). Thus, our findings indeed demonstrated that FOXM1induces metastasis of gastric cancer through induction of Cath-D.

### E-cadherin is downregulated by Cath-D

**Figure 9:**
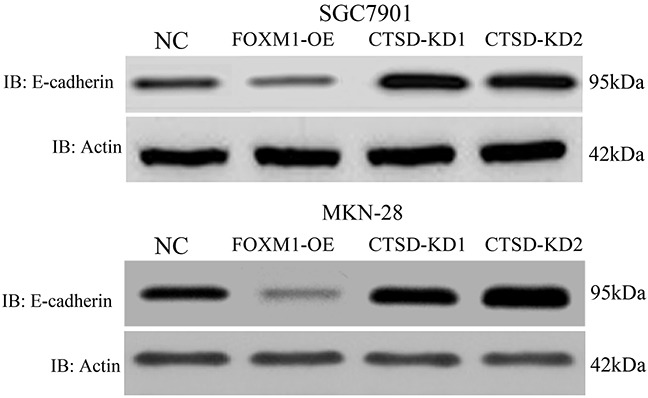
E-cadherin expression in SGC7901 and MKN28 with cathespin L knockdown or overexpression We have shown that Cath-D expression is elevated in FOXM1-OE cells, and E-cadherin expression is suppressed in these cells. In CTSD-KD cells, E-cadherin expression is elevated. This suggested a negative correlation between Cath-D and E-cadherin in SGC7901 and MKN28 cells.

As reported, cleavage of E-cadherin needs the help of Cath-D, which affects cell-cell adhesion and induces EMT in pancreases cancers. To further validate the molecular mechanisms underlying Cath-D-induced metastasis in gastric cancer, we detected the expression of E-cadherin in FOXM1-OE and FOXM1-OE-Cath-D-KD cells using western blotting analysis. As illustrated in Figure 9, the overexpressed Cath-D in FOXM1-OE cells down-regulated the expression of E-cadherin. Conversely, knockdown of Cath-D in FOXM1-OE cells enhanced the expression of E-cadherin. These findings suggested that Cath-D cleaves E-cadherin to promote EMT of gastric cancer cells in the progression of gastric cancer

### FOXM1 activates Cath-D promoter

**Figure 10:**
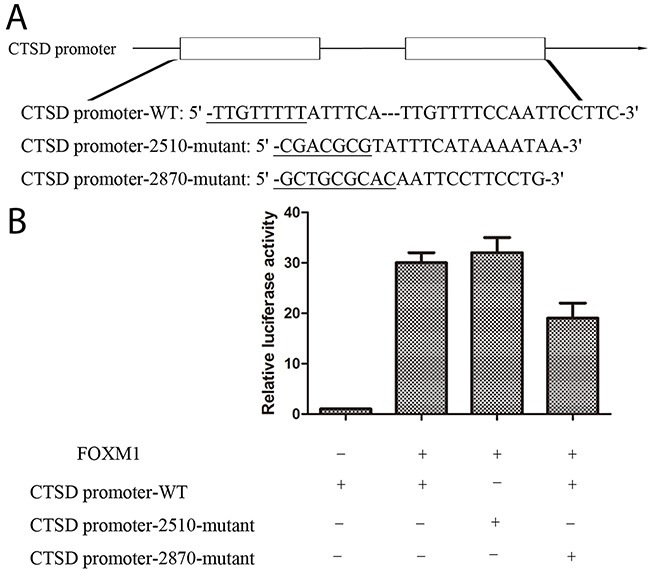
FOXM1 activates Cath-D promoter **(A)** pGL3-FOXM1 was transfected into MKN28 cells previously transfected with CTSD promoter-WT or CTSD promoter-2510-mutant or CTSD promoter-2870-mutant constract. **(B)** Luciferase assays were performed to measure activity levels of three Cath-D promoters. Three independent experiments were carried out to obtain standard deviation.

To validate whether Cath-D serves as a transcriptional target of FOXM1, three Cath-D promoter fragments (the wild type or mutant FOXM1 putative target site) were cloned into pGL3-control vector. As shown in Figure 10, we observed that luciferase activity was obviously decreased in cells with cotransfection of Cath-D promoter-2870-mutant and pGL3-FOXM1as compared with Cath-D promoter-WT and pGL3-FOXM1, indicating that FOXM1targets −2870 site of Cath-D promoter to affect the transcriptional expression of Cath-D.

## DISCUSSION

To date, metastasis has become a major factor of gastric cancer-caused mortality, the molecular mech-anisms underlying cancer cell metastasis are still unclear. In this study, we identified FOXM1has an invasive role in the progression of human gastric cancer. Firstly, our observations showed that the expression of FOXM1 was obviously higher in gastric cancer tissues compared with that in adjacent non-tumor tissues [16]. Secondly, cell function analyses demonstrated thatFOXM1overexpression of FOXM1induced cell migration and invasion in gastric cancer. These findings indicated that FOXM1might acts as a tumor promoter to participate in metastasis of gastric cancer cells.

Next, luciferase assays were used to selected candidate targets of FOXM1, which were associated with cell migration and invasion. It has been reported that degradation of ECM components plays an important role in gastric cancer invasion and metastasis. Notably, Cath-D has been identified to be a family of cysteine proteases, and is able to degrade a majority of the ECM components when secreted into the extracellular environments. As reported by Bach AS et al, Nuclear cathepsin D enhances TRPS1 transcriptional repressor function to regulate cell cycle progression and transformation in human breast cancer cells [17]. In addition, human glioblastoma transfected with Cath-D exhibited stronger invasion than control [18]. Thus Cath-D,contributes to invasion of cancer cells into the surrounding tissues andvessels and even distant organs [19, 20]. In the present study, we carried out luciferase assays and demonstrated that luciferase activity was significantly increased when cells were cotransfected with FOXM1 and a Cath-D3′-UTR. Consistent with luciferase assay, the expression of Cath-D protein was also positively associated with the expression of FOXM1 in gastric cancer tissues, and its expression was also obviously decreased in FOXM1-knockdown SGC7901 and MKN28 cells, suggesting that Cath-D is a direct target of FOXM1. It should be noted that Cath-D also decreased cell-cell adhesion by the cleavage of E-cadherin [21]. In this work, FOXM1-overexpression increased expression levels of Cath-D to decrease the expression of E-cadherinin gastric cancer cells. Because Cath-D is not related tocell proliferation [22], we found cell growth in SGC7901 and MKN28 with Cath-D knockdown cells was not altered compared with the control siRNA cells.

In this study, FOXM1 induces cell migration and invasion of gastric cancer by inducingCath-D expression. However, current reports showed that the increase ofFOXM1 expression might predict a good prognosis in gastric cancer patients [18, 23]. According to recent references, we assumed that FOXM1 plays a different role in the progression of gastric cancer in different contexts, and FOXM1 also exert diverse biological effects at different stages. In the early stage, FOXM1 may be inactivated, which contributes to cancer cell proliferation. However, in later stages, FOXM1 activation will promote cancer cell migration and invasion [24]. In the next study, we will carry out further studies to identify whether FOXM1 is modulated by other transcription factors, in the progression of gastric cancer. Thus, FOXM1 may be also a risk factor in gastric cancer patients with radiation or chemotherapy.

In conclusion, this study demonstrated that FOXM1 promotes gastric cancer cell migration and invasion by inducing Cath-D, and then Cath-D cleaves E-cadherin to induce EMT development. Thus, our study indicated that FOXM1 and Cath-D might be potential and useful targets for gastric cancer patients, especially patients with metastasis.

## MATERIALS AND METHODS

### Ethical statement

All procedures performed in studies involving human participants were in accordance with the ethical standards of the institutional and/or national research committee and with the 1964 Helsinki declaration and its later amendments or comparable ethical standards.

### Cell lines and cell cultures

Gastric carcinoma cell lines SGC7901 and MKN28 were purchased from the Shanghai Cell Bank of the Chinese Academy of Sciences (Shanghai, China). 293T cells were purchased from American Tissue culture collection (ATCC). All cells were cultured in RPMI 1640 (HyClone, Logan, Utah, USA) supplemented with 10% FBS in a 5% CO_2_ humidified atmosphere at 37°C.

### Tissue samples

20 paired GC and corresponding adjacent nontu-morous samples were obtained from patients who underwent surgery at The Second Affiliated Hospital of Dalian Medical University between 2014 and 2015. All cases were confirmed as GC based on histopathological evaluation. The clinicopathological characteristics of the GC patients were summarized. No local or systemic treatment was conducted in these patients before surgery. All collected tissue samples were immediately snap-frozen in liquid nitrogen and stored at −80°C until required. Our study was approved by the Research Ethics Committee of The Second Affiliated Hospital of Dalian Medical University. Written informed consent was obtained from all patients.

### Lentivirus-delivered shRNA gene knockdown

Lentivirus-delivered shRNA gene knockdown was performed. Briefly, the shRNA sequences used for lentiviral silencing FOXM1 were 5′-GCTCTTGGTGGA TCATCAA-3′ and 5′-CATGTTCAATGGGAGCTTGGA-3′. The Cath-D silencing sequences were 5′-GGCGATGCACA-ACAGATTA-3′ and 5′-TGACACCGGCTTTGTGGAC-3′. The shRNA sequences were cloned into the pLenti lentiviral vector (Hanyin Co., Shanghai, China). The respective lentiviral vectors and packaging vectors were transfected into 293T cells for viral packaging. Sixty hours after transfection, the virus was collected to infect target cells in the presence of 8mg/ml polybrene (Sigma-Aldrich). Then, independent stable clones were selected and evaluated by western blotting.

### Retrovirus-mediated gene expression

FLAG-tagged FOXM1 was cloned into the pMSCV-IRES-GFP vector. For FOXM1 overexpression, cells were infected with viral supernatants from 293T cells transfected with FOXM1 or the control MSCV.

### Wound-healing assay

For wound-healing assays, cultured cells were resuspended and seeded in 6-well plates. A 200 μl pipette tip was used to make a scratch across the plate to form an artificial wound. Detached cells or cell debris were washed away after scratching. After 24, 48 and 72 hours, images of the wound areas under each condition were photographed. The percentage of wound closure was measured by the following formula (1-[current wound size/initial wound size])*100%.

### Invasion assay

Invasion assays were performed using Transwell chambers with 8μm-pore size polycarbonate membrane filters. The upper surface of the filter was coated with Matrigel (Becton Dickinson, Bedford, MA). The Matrigel was dried at 37°C for 2 hours and placed at room temperature overnight. The cells were starved overnight, resuspended and seeded in the upper chamber at a density of 5 × 10^4^ cells/well. After 24 hours of transfection, the cells that invaded the lower surface of the filter were fixed and stained. Cells were counted in 10 randomly selected fields under a light microscope at high magnification. Experiments were repeated at least in triplicate.

### MTT assays

Cell viability was tested with a Cell Proliferation Reagent Kit I (MTT) (Roche Applied Science). The cells were grown in 96-well plates. Cell viability was assessed every 24h following the manufacturer’s protocol. All experiments were performed in quadruplicate.

### Western blotting

Proteins were extracted from log-phase cells and dissolved in 2X SDS sample buffer. Protein concentrations were quantified using a BCA protein assay kit (Pierce, Rockford, IL). After electrophoresis, the proteins were separated and transferred to nitrocellulose membranes and blocked with nonfat milk. The membrane was incubated with the appropriate primary antibody (FOXM1 1:1000 dilution, Cath-D 1:1000 dilution) for 1 hour as previously described [12]. The blot was washed three times with PBS, incubated with streptavidin-peroxidase for 15 min, and developed by the enhanced chemiluminescence method (Sigma, St. Louis, MO).

### Immunohistochemical analysis

Twenty cases of tissue microarray blocks were immunostained for FOXM1 and Cath-D proteins and analyzed. Briefly, immunohistochemistry was conducted using a highly sensitive streptavidin-biotin-peroxidase detection system with FOXM1 monoclonal antibody (1:100 dilutions) and Cath-D antibody (1:100 dilution). We quantitatively scored the tissue sections according to the percentage of positively stained cells as well as staining intensity.

### Luciferase reporter assay

Bioinformatics analysis was used to predict binding regions for FOXM1 on promoter of Cath-D. Three segments of the promoter of Cath-D (Cath-D promoter-WT: 5′-TTGTTTTTATTTCATTGTTTTCCA ATTCCTTC-3′, Cath-D promoter-2510-mutant: 5′-CGAC GCGTATTTCATAAAATAA-3′, Cath-D promoter-2870-mutant: 5′-GCTGCGCACAA TTCCTTCCTG-3′) were cloned into pGL3-basic-Report (Promega, WI, USA) as well as human 3′-UTR of the FOXM1 gene. The reporter vectors containing the promoters of Cath-D and pGL3-FOXM1 were transfected into MKN28 cells. After 48 h, luciferase activity was measured with a dual-luciferase reporter assay system (Promega, WI, USA).

### *In vivo* tumor metastasis assays

Sterile PBS (0.2 ml) containing approximately 1 × 10^6^ SGC7901 cells were injected into the tail veins of male nude mice. The mice were then monitored for overall health and body weight. Eight weeks after the injection, the mice were sacrificed, and their lungs were isolated. After counting the number of visible tumors on the lung surface, serial sections of the liver tissues were made and observed using imaging microscopy. There were 6 mice in each group.

### Statistical analyses

Correlation analyses were performed using the two-sided χ2 test or Fisher's exact test. Two-tailed Student's t tests and one way ANOVA were used to analyze differences between the protein overexpression and knockdown groups from *in vitro* experiments. A P-value <0.05 was considered statistically significant.
